# The bright side of fibroblasts: molecular signature and regenerative cues in major organs

**DOI:** 10.1038/s41536-021-00153-z

**Published:** 2021-08-10

**Authors:** Rita N. Gomes, Filipa Manuel, Diana S. Nascimento

**Affiliations:** 1 Instituto de Investigação e Inovação em Saúde (i3S), Universidade do Porto, Porto, Portugal; 2 Instituto de Ciências Biomédicas Abel Salazar (ICBAS), Universidade do Porto, Porto, Portugal; 3 Instituto Nacional de Engenharia Biomédica (INEB), Universidade do Porto, Porto, Portugal; 4 Faculdade de Ciências, University of Lisbon, Lisbon, Portugal

**Keywords:** Mechanisms of disease, Ageing, Diseases

## Abstract

Fibrosis is a pathologic process characterized by the replacement of parenchymal tissue by large amounts of extracellular matrix, which may lead to organ dysfunction and even death. Fibroblasts are classically associated to fibrosis and tissue repair, and seldom to regeneration. However, accumulating evidence supports a pro-regenerative role of fibroblasts in different organs. While some organs rely on fibroblasts for maintaining stem cell niches, others depend on fibroblast activity, particularly on secreted molecules that promote cell adhesion, migration, and proliferation, to guide the regenerative process. Herein we provide an up-to-date overview of fibroblast-derived regenerative signaling across different organs and discuss how this capacity may become compromised with aging. We further introduce a new paradigm for regenerative therapies based on reverting adult fibroblasts to a fetal/neonatal-like phenotype.

## Introduction

Tissue damage can have several causes, including mechanical forces, infections, toxins, ischemia, and autoimmune reactions^1^. In an ideal scenario, the response to injury is able to restore normal organ architecture and function, a process that is known as regeneration^2^. Independently of the organ, regeneration usually comprises three overlapping phases. Immediately after injury, apoptotic cells at the injury site signal toward macrophage and neutrophil recruitment for clearance of cell debris and avoid infection, thus initiating the inflammatory stage. From 2 to 10 days after injury, the proliferative stage begins and processes such as angiogenesis, extracellular matrix (ECM) deposition, and cell proliferation create new tissue and reduce the injured area^2^. Finally, the remodeling stage takes place, in which the tissue recovers the preceding organization and the underlying ECM is reorganized^3^. Dysregulation of any of these processes can trigger excessive ECM deposition, typically rich in collagen I, resulting in the formation of scar tissue—a process that is widely known as fibrosis. The latter is therefore associated to organ repair and negatively impacts organ function^4^.

Fibroblasts are cells of mesenchymal origin and are the main producers of ECM in homeostatic conditions and in response to injury^5^. These cells are found in virtually every tissue, but their molecular signature is not preserved between organs^6^. Fibroblasts are activated in the inflammatory stage of the wound healing in response to cytokines and growth factors such as transforming growth factor beta 1 (TGF-β1)^7^, interleukin 1 beta (IL-1β)^8^, interleukin 6 (IL-6) or platelet-derived growth factor (PDGF)^9^, and differentiate into myofibroblasts^5^. Less characterized cells like pericytes, mural cells typically associated with endothelial cells, are also capable of differentiating into myofibroblasts^10^. The latter display distinctive features such as increased cell size, high alpha smooth muscle actin (α-SMA) expression, and the presence of microfilaments that supports cell contraction. Importantly, myofibroblasts secrete great amounts of ECM and are therefore regarded as the culprits of fibrotic diseases and organ dysfunction after injury^2^. Current strategies to treat or reverse fibrosis focused on targeting myofibroblasts include inducing apoptosis or senescence, promoting dedifferentiation to fibroblasts and reprogramming into other cell types^11^. Recently, the use of chimeric antigen receptor (CAR)-T cells for targeting activated cardiac fibroblasts has shown great potential to reduce myocardial fibrosis supporting immunotherapeutic strategies as a new avenue to control fibrosis^12^. Yet, little anti-fibrotic therapies are effective and currently available in the clinical setting^1^.

Considering the well-described contribution of fibroblasts in the development of fibrosis, their role in other biological events is often neglected. However, highly regenerative organs like the intestine rely on fibroblast activity to maintain the stem cell niche^13^. In other organs like the liver or the lung, where regenerative mechanisms encompassing the proliferation of differentiated cells or progenitor cell differentiation are only activated after injury, fibroblasts are able to secrete growth factors and mitogens and produce structural components of ECM to restore normal tissue architecture^14,15^. Even in a non-regenerative organ like the heart, where fibroblast activity is mainly associated to fibrosis and organ dysfunction, neonatal fibroblasts and some ECM components have been shown to promote cardiomyocyte proliferation after injury^16^. Hence, one can hypothesize that fibroblasts in most organs are actively involved in repair as well as in regeneration. This review will focus on the characterization of fibroblast signatures in different organs and unveil potential targets for stimulating fibroblast-induced regeneration in wound healing and fibrotic diseases.

## Organ-specific fibroblasts: surface markers and regenerative signals

### Intestine

**Fig. 1:**
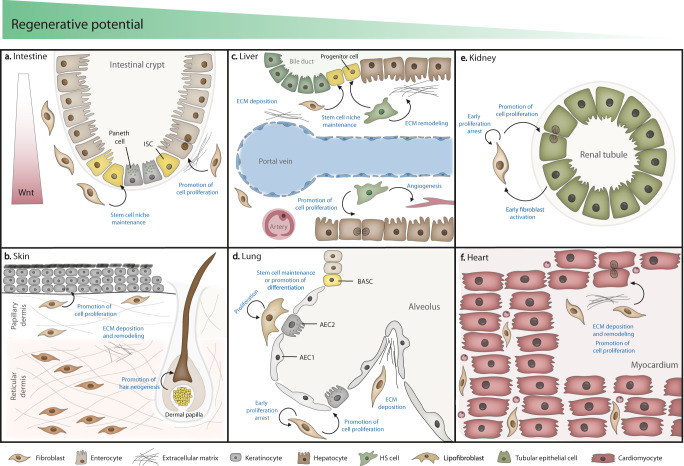
The fibroblast as a mediator of regeneration in major organs. **a** Intestinal fibroblasts are a component of the stem cell niche, secreting ECM, growth factors, and creating a Wnt gradient along the crypt promoting ISC proliferation/differentiation. **b** Dermal fibroblasts promote regeneration mainly by secreting specific ECM components, promoting wound healing. A specialized population of fibroblasts (dermal papilla) is also involved in hair neogenesis. **c** In the liver, portal fibroblasts and HS cells promote liver progenitor and hepatocyte proliferation after injury by multiple mechanisms. **d** Proliferative lung fibroblasts and lipofibroblasts promote regeneration by secreting structural ECM components and stimulating AEC2 cells with mitogens. **e** In the kidney, fibroblasts and epithelial cells communicate in a bidirectional fashion to coordinate tubular regeneration. **f** Specific ECM components putatively secreted by fibroblasts have been linked to cardiac regeneration in the neonatal heart.

The intestine is an organ which self-renews its lining every few days, much owing to the presence of intestinal stem cells (ISC) in the crypts^17^. Fibroblasts, along with other cells, encompass a supporting niche for leucine-rich repeat-containing G-protein 5 (Lgr5)^+^ ISC^18,19^ (Fig. 1a) and were found to express GLI family zinc finger 1 (GLI-1), podoplanin, CD90, vimentin, and fibroblast-specific protein 1 (FSP-1)^20–22^. Some cells express α-SMA in physiological conditions, and are considered by some to be myofibroblasts^23^. Unsupervised clustering gene expression analysis also showed that human gastrointestinal fibroblasts segregate from fibroblasts isolated from other organs^24^. The major differences in gene expression concerned upregulation of some transcriptional regulators (e.g., *Tcf21*, *Foxf1*, *Foxp2*), signaling molecules (e.g., chemokine ligands, fibroblast growth factors), and ECM remodeling genes (e.g., *Comp*, *Col3a1*, *Lama3*).

The maintenance of the ISC niche by intestinal fibroblasts appears to be mediated by the establishment of a Wnt/BMP gradient, where Wnt signaling is the main driver of crypt proliferation. At the base of the crypt where ISC reside, fibroblasts secrete Wnts and BMP antagonists^25,26^. In a single-cell RNA-sequencing approach targeted for human intestinal CD90^+^ cells, the authors identified 11 clusters from which two were crypt niche cells^27^. Besides expressing the conventional fibroblast markers vimentin, collagen I, and collagen III, these cells also expressed noncanonical Wnt ligands, BMPs, and periostin. Gene ontology analysis showed enriched terms of “response to wound healing” and “regulation of epithelial cell proliferation.” In the same report, these cells were decreased in the biopsies of inflammatory bowel disease patients, who present exacerbated intestinal inflammation and decreased mucosal healing, which reveals a role of these cells in supporting epithelial renewal^28^. Furthermore, PDGFRα^+^ fibroblasts have been shown to secrete Wnt and R-spondin 3 in the pericryptal region^29^. R-spondin 3 acts as a Wnt enhancer and is vital to injury repair by inducing more differentiated Lgr4^+^ cells to regain Wnt production, and thus generate new crypts^29,30^. Gli1^+^ gp38^+^ colonic fibroblasts were also found to express *Wnt2b* and thus to be possible stem cell niche-supporting cells^21^. These cells were later found to be pericryptal CD90^+^ fibroblasts, capable of supporting endothelial cell proliferation and organoid growth in vitro. Subsequent differential gene expression analysis unveiled upregulated expression of stem cell niche factors, hepatocyte growth factor (HGF), and class 3 semaphorins (Sema III). Of note, inhibiting the binding of semaphorins to their receptors prevented CD90^+^ fibroblasts-mediated organoid growth. Altogether, these reports indicate that fibroblasts are key drivers of ISC maintenance and crypt proliferation through Wnt/BMP signaling. In line with this, age-associated decrease in regenerative ability has been correlated with a reduction in Wnt signaling, and exogenous Wnt administration rescued the aged ISC phenotype in an organoid model^31,32^.

Other reports suggest that these cells also directly promote epithelial cell proliferation by secreting HGF^33^ and expressing periostin^34^. Furthermore, in an injury setting, the inflammatory milieu sensed by tumor progression locus-2 (Tpl2) kinase-expressing subepithelial myofibroblasts triggers a compensatory epithelial proliferation via ERK, cycloxygenase 2 (Cox-2), and prostaglandin (PGE_2_)-activated signaling—a response, which is dysregulated in inflammatory bowel diseases^35^. In a similar fashion, the prostaglandin signaling pathway in Cox-2^+^ fibroblasts was found to positively influence stem cell antigen-1 (Sca-1)^+^ ISC expansion, and trigger epithelium regeneration or tumor formation, in case ISC underwent previous mutations^36^. Overall, intestinal fibroblasts directly stimulate cell proliferation and are critical for supporting the stem cell niche and epithelial renewal, a capacity that decreases with aging thus contributing toward a decline in the regenerative capacity of the organ in the elderly.

### Skin

The skin is the largest organ in the body and a complex structure comprising the epidermis, dermis, hair follicles, and other appendages. In homeostasis, basal epidermal stem cells ensure the re-epithelization after normal skin shedding or after insults to the outermost layer of the skin, the epidermis^37^. Fibroblasts stimulate keratinocyte proliferation and differentiation in the epidermis mainly by the release of soluble factors such as IL-1, keratinocyte growth factor (KGF), and granulocyte-macrophage colony-stimulating factor (GM-CSF) as shown in studies using dermal equivalents with epidermal keratinocyte/fibroblast co-cultures^38–40^. Yet, the dermis, the thickest layer of the skin, is the most studied layer in wound healing^37,41^. Rognoni et al. postulate that an initial phase of active dermal fibroblast migration and proliferation followed by high ECM deposition phase, which negatively regulates fibroblast proliferation, is key to successful wound healing^42^. Dermal fibroblast progenitors expressing Twist-related protein 2 (Twist2/Dermo1), platelet-derived growth factor receptor α (PDGFRα) and Engrailed1 (En1) give rise to papillary fibroblasts and to reticular fibroblasts (as reviewed in ref. ^43^) which reside in the upper papillary dermis and the lower reticular dermis, respectively (Fig. 1b), and display a distinct signature. In the human skin, mutually exclusive CD90 and fibroblast activation protein (FAP) expression are enough to discriminate both populations^44^. Papillary and reticular fibroblasts influence the composition of the respective layer of the dermis. Well-organized fibrillary collagen bundles are abundant in the reticular dermis, while non-fibrillary collagens and proteoglycans like fibromodulin and decorin are more common in papillary dermis^41,45^. This suggests that these populations directly influence the ECM components of the dermis and may respond differently to injury. Jiang and Rinkevich reviewed the subsets of dermal fibroblasts implicated in fibrosis and concluded that no markers or spatial location within the dermis can discriminate fibrotic fibroblasts^46^. Conversely, other evidences point that papillary fibroblast favor scar-free wound healing by producing Wnt whereas reticular fibroblasts are fibrotic since readily synthetize collagenous ECM^47,48^. In fact, keratinocytes grown on papillary dermis-like ECM proliferate more than on reticular fibroblast-derived ECM^49^. In addition, reticular and papillary fibroblasts have been described to respond differently to aging^50,51^. In a comparative study, aged papillary fibroblasts display reduced capacity to proliferate and remodel ECM relatively to aged reticular fibroblasts^51^. The authors of this study suggest that the less-differentiated papillary fibroblasts may progressively disappear or differentiate into reticular fibroblasts with aging. These evidences collectively suggest that papillary fibroblasts are more pro-regenerative than reticular fibroblasts and that their stimulation can be key for regenerating skin without scarring^52^.

Aiming at understanding the mechanisms governing scarless skin regeneration, significant interest has been given to mammalian fetuses as they are able to regenerate their skin without scarring^53–55^. The fetal healing process was found to differ from the one of adults in various parameters such as inflammatory cell recruitment, TGF-β expression, and ECM secretion^54,56^. In fetal wound healing, ECM components like collagen III, hyaluronic acid, and matrix metalloproteinase (MMP) to inhibitor (TIMP) ratio are upregulated, which is thought to favor cell migration^54^. Mouse neonates can also regenerate their skin. The loss of regenerative potential within the dermis as mice age is correlated to a decrease in fibroblast proliferation, particularly reflected in the cellular density of the papillary layer, and a decrease in Wnt signaling^57^. A single-cell RNA-sequencing study comparing skin in developing (P2), regenerating (wounded P2), homeostatic (P21), scarring (wounded P21) conditions has unveiled that pro-regenerative fibroblasts are papillary fibroblasts expressing *Lef1*^58^. The reactivation of *Lef1* in adult animal dermis enhanced wound healing.

One of the few examples of adult human scar-free healing occurs in injuries of the oral mucosa. Here, the microenvironment is less inflammatory and fibroblast-secreted ECM is rich in ED-A form of fibronectin, chondroitin sulfate and has less elastin^59^. The authors postulated that chondroitin sulfate promotes faster wound closure. On the other hand, fibronectin ED-A, which is typically produced by fibroblasts, has been shown to promote a normal wound healing process likely by promoting epithelial cell migration^60^. An independent study showed that human oral mucosa fibroblasts are reportedly more prone to express glycoproteins and transcription factors that promote angiogenesis, cell migration and proliferation, and less prone to the expression of senescent markers than human dermal fibroblasts^61^.

Animal models with enhanced capacity to regenerate are important models to dissect regenerative mechanisms. Recently, a small mammal able to regenerate the skin and hair has been identified. *Acomys*, the spiny mouse, is a rodent whose skin thickness is very similar to *Mus musculus* but tears easily at the back or in the tail^62^. After injury, almost no α-SMA^+^ myofibroblasts are found at the lesion site^62^. The composition of the ECM is different in *Acomys* wounds and molecules like collagen triple helix repeat containing-1 (CTHRC-1), tenascin-C, fibronectin 1, laminin α1, and aggrecan are upregulated and correlated to a regeneration-inducing environment, although no mechanistic details have been attributed to these components individually^63^. However, CTHRC-1 has been described as a promoter of wound closure by recruiting anti-inflammatory macrophages^64^. Therefore, the underlying ECM may be responsible for regeneration in *Acomys* wounds. In fact, Brant et al. suggested that a low inflammatory environment, due to lesser induction of cytokines and chemokines, combined with the presence of fetal-like ECM may underlie scarless regeneration in this animal^65^. This evidence further supports fetal fibroblasts display a pro-regenerative phenotype.

Another challenge of skin regeneration is the restoration of hair follicles. Most adult mammals do not fully restore these structures after injury but the opposite has been demonstrated in full thickness wounds in mice and rabbits^66,67^. Hair follicle neogenesis requires coordination between stem cells that generate all components of the follicle and the dermal papilla niche, which comprises specialized fibroblasts derived from the papillary lineage^68,69^. Dermal papilla cells usually express CD133 and alkaline phosphatase^70^. In mice, Blimp1^+^ fibroblasts contribute to hair follicle formation through Wnt/β-catenin signaling activation in the dermal papilla^71^. Moreover, activation of Sonic hedgehog (Shh) in wound fibroblasts has been also correlated to dermal papilla formation and hair growth stimulation^72^. Hence, the stimulation of fibroblast-mediated Wnt or Shh signaling may pose as a promising strategy for hair follicle renewal.

In the skin, fibroblasts have been shown to influence dermal regeneration by secreting specific ECM components (reviewed elsewhere^73^) and, in the case of hair production, fibroblasts are also essential for regulating the stem cell niche. Of note, scarless free skin regeneration is restricted to fetal stages or to specific environments, such as the oral mucosa, in which fibroblast acquire a fetal-like phenotype. In adulthood, papillary fibroblasts have been shown to signal toward regeneration but further studies are required for unveiling involved mechanisms.

### Liver

The two aforementioned organs, the skin and intestine, are epithelial organs with high cell turnover, in which cell renewal and tissue homeostasis are achieved through activation of a tissue-specific pool of stem/progenitor cells. Instead, the liver has low cell turnover but regenerates in a unique manner. Following an up to two-thirds mouse liver resection, the organ becomes fully recovered in ~2 weeks^74^. This efficient replacement is mainly at expenses of adult mature hepatocyte self-renewal, and to a lesser extent granted by the existence of progenitor cells^74–76^.

Portal fibroblasts and hepatic stellate cells (HS) or sinusoidal pericytes are the main populations of resident mesenchymal cells in the liver^77^ (Fig. 1c). At steady state, HS are in contact with hepatocytes and sinusoids, and portal fibroblasts are found surrounding the portal vein and bile ducts in the portal region. Despite some phenotypic heterogeneity, both are recognized by the expression of specific markers (reviewed in refs. ^78,79^). Single-cell RNA-sequencing approaches using mesenchyme-labelling PDGFRβ expression enabled the transcriptomic and spatial in-depth characterization of different subsets of fibroblasts and HS, both in healthy and injured livers^80,81^. Dobie et al. describe a population with upregulated ECM pathways (portal fibroblasts) and a population with upregulated vitamin A metabolism processes (HS). After centrilobular necrosis injury, a subset of lysophosphatidic acid receptor 1-positive (LPAR-1) HS is the main culprit for collagen secretion and fibrosis. Krenkel et al. find four subpopulations of myofibroblasts derived from HS in the injured liver, with the surface marker S100 calcium binding protein A6 (S100A6) being expressed in all of them. Indeed, HS appear to be the main source of myofibroblast-like cells following most injuries, having a central role in hepatic fibrosis and the underlying imbalance between ECM synthesis and degradation^82^. Portal myofibroblasts also play a part in liver fibrosis, with a particular role in biliary diseases^83^.

Interestingly, both HS and portal myofibroblasts can play a role in liver regeneration. What makes these cells pro-regenerative or pro-fibrotic is still not known, although some authors hypothesize that this response lies on yet to be described subpopulations^15^. Activated HS undergo changes such as the loss of vitamin A and upregulation of α-SMA, and become capable of releasing mitogens, like HGF, pleiotrophin, and epimorphin, to stimulate hepatocyte proliferation^84^. Activated HS also influence the recruitment of immune cells and angiogenesis by vascular endothelial growth factor (VEGF) and erythropoietin secretion^85^, and induce ECM remodeling via secretion of MMP-1, MMP-2, and angiopoietin^15,86^. HS promote the proliferation of progenitor cells through the secretion of IL-6, fibroblast growth factor 7 (FGF-7), lymphotoxin-beta, and Hh, as reviewed in ref. ^87^. HS have also been shown to become progenitor cells and support liver regeneration in a Hedgehog-regulated fashion after hepatectomy^88^. HS cells first differentiate into a myofibroblast phenotype to then repopulate the liver with newly formed hepatocytes. In parallel, portal fibroblasts also appear to promote survival of progenitor cells in the bile duct region via Hedgehog^89^ and by losing nucleoside triphosphate diphosphohydrolase-2 (NTPDase2) expression^90^. Furthermore, portal fibroblasts in the regenerating rat liver express neural cell adhesion molecule (NCAM), an adhesion molecule that associates with collagens and provides binding sites to other cells, ensuring regrowth of portal tracts^91^.

Apart from contributing for both liver repair and regeneration after injury, HS have a role in reverting fibrosis. The concept of liver fibrosis reversal has been addressed in humans and animal models^92^ and has even been confirmed in cases of hepatitis after antiviral treatment^93^. Activated HS are either eliminated by apoptosis, become senescent, and later cleared by NK cells, or survive but become inactivated^94–96^. If HS become apoptotic, TIMP-1 production decreases and restitution of homeostatic MMP/TIMP ratio impedes buildup of new ECM and the existent collagenous matrix is degraded^97–99^. If HS become inactivated, fibrotic genes like *Spp1*, *Col1a1*, and *Acta2* (α-SMA) are downregulated and activated HS acquire characteristics similar to those found in the quiescent counterparts^95,100^.

Changes in ECM after injury have also been shown to influence liver regeneration. For example, vitronectin and olfactomedin 4 (OLFM4) production in injured human livers promote cell migration and adhesion, while fibronectin and collagen I induce cell proliferation and differentiation of liver progenitors into hepatocytes^101^. MMP-2 and -9 are also key players in liver regeneration, as they affect ECM remodeling and the release of HGF and other growth factors^102,103^. Importantly, a decrease in ECM remodeling via MMP activity and subsequent increase in fibrosis is correlated with aging^104^.

In the liver, the same cells involved in fibrotic response can signal also for regeneration for which ECM composition and remodeling appears to be crucial. Yet, further studies are needed to pinpoint cellular subpopulations that may contribute mostly to regeneration instead of repair.

### Lung

Alike the liver, the lung presents limited cell turnover in homeostasis. However, this organ has been proven to regenerate after injury (i.e., facultative regeneration) owing mostly to a pool of stem cells. The latter exit their quiescent state^105^, namely bronchioalveolar stem cells (BASC) that are able to originate both bronchiolar and alveolar epithelial cells^106^, and alveolar type II cells (AEC2) in alveoli^107^, where gas exchanges occur and the organ is most susceptible to external insults.

Surrounding alveoli are resident lung fibroblasts (Fig. 1d), which express PDGFRα, collagen I, CD146, vimentin, and desmin (reviewed in ref. ^108^). These cells contribute to the formation of an ECM that supports alveolar regeneration. For example, in vitro and in vivo studies show that elastin and collagen secreted by lung fibroblasts aid alveologenesis after birth by providing support to alveoli septation^109,110^. Fibroblast-secreted fibronectin is also important for endothelial cell adhesion, as shown in an in vitro study using fibroblast-derived matrices^111^.

Because PDGFRα was found to be important for alveolarization during development and realveolarization^112^, Endale et al. have recently traced PDGFRα^+^ fibroblasts spatial location throughout development^113^. At postnatal stages, these fibroblasts, which include subpopulations of myo and lipofibroblasts, co-localized with alveoli. In addition, in the human lung, a single-cell RNA-sequencing study unveiled the spatial distribution of at least eight stromal populations, and alveoli were found to be surrounded by myofibroblasts, alveolar fibroblasts, and lipofibroblasts^114^. Lipofibroblasts are best characterized by the expression of adipose differentiation-related protein (ADRP), peroxisome proliferator-activated receptor γ (PPARγ), parathyroid hormone 1 receptor (PTH1R)^115^, and transcription factor 21 (Tcf21)^116^. These cells are lipid-containing interstitial fibroblasts that are found in close contact with AEC2 cells and aid in surfactant production in alveoli^117^. Barkauskas et al. have proposed that AEC2 are adult stem cells, which are directly supported by PDGFRα^+^ lipofibroblasts in the formation of alveolospheres in vitro by stimulation of AEC2 proliferation and differentiation^107^. In fact, PDGFRα^+^ fibroblasts directly influence AEC2 regeneration after injury by proliferating and secreting IL-6, FGF-7, and BMP inhibitors^118^. A single-cell RNA-sequencing approach confirmed a role of alveolar lipofibroblasts in fibrosis^119^, although retinoic acid-metabolizing lipofibroblasts are also regarded as pro-regenerative cells after a seminal study demonstrated that retinoic acid administration induced regeneration in a rat emphysema model^120,121^. Retinoic acid, a bioactive metabolite of vitamin A, induces changes in gene expression by binding to specific transcription factors and has been previously linked to regeneration of urodele limbs, central nervous system, and lungs^122^.

Some fibroblast populations also signal directly for epithelial regeneration after pneumonectomy. For example, Fsp1^+^ fibroblasts are beneficial to regeneration if their proliferation is transient and contained to the first few days after injury when IL-6 and cell-cycle genes are upregulated^123^. PDGFRα^+^ fibroblasts increase in realveolarization after pneumonectomy^112^ and are the main source of KGF and HGF, which induce epithelial and endothelial proliferation after injury^124^. Noteworthy, the response to pneumonectomy seems to change with age. Compared to 3-month-old, 9-month-old murine fibroblasts have lower ability to proliferate in vitro and adopt a myofibroblastic phenotype^125^.

In sum, fibroblasts contribute to lung regeneration mainly by proliferating in early stages of injury, whilst secreting mitogens and structural ECM components. Yet, the influence of ECM composition in lung regeneration remains unclear as has been mainly addressed in vitro. This response changes with age, since the proliferative capacity of fibroblasts decreases in older animals, demonstrating the key role of this cell type orchestrating regeneration in this organ.

### Kidney

The kidney is an organ with limited regenerative potential since quiescent tubular epithelial cells regain capacity to proliferate after acute injury but structural regeneration of the nephron—the functional unit of the kidney—is not achieved^126,127^.

Resident fibroblasts are sparsely dispersed between the tubules and peritubular capillaries (Fig. 1e). They express vimentin but not desmin^128^, FSP-1, cadherin-9^129^, and secrete erythropoietin to maintain homeostatic conditions in response to hypoxia^130^. Kidney-resident fibroblasts and pericytes express PDGFRβ and CD73, and derive from the same progenitor, suggesting that these populations are likely overlapping^131^, although the subject is still controversial^132^.

After acute kidney injury, kidney fibroblasts are able to promote tubular regeneration^133–135^. The latter depends on the bidirectional communication between tubular epithelial cells and fibroblasts^133^. The injured epithelium promotes early fibroblast activation by releasing Shh and TGF-β1-containing exosomes^136,137^. Then, activated fibroblasts promote tubular repair via Wnt/β-catenin signaling, retinoic acid production, and HGF secretion^138–140^. Apart from stimulating epithelial proliferation, HGF also inhibits TGF-β signaling in fibroblasts, preventing their further activation and fibrosis^140^. In fact, early fibroblast proliferation in acute kidney injury is required for regeneration^137^. In acute injuries, initial fibroblast/myofibroblast expansion regresses after regeneration, contrarily to what is observed in chronic injuries, in which regeneration is not present. This seems to relate with the downregulation of genes related to ECM organization, TGF-β, and MMP signaling in fibroblasts during the resolution phase of acute injuries whereas in chronic scenarios, these genes remain upregulated. In addition, expression of inflammation-associated molecules in fibroblasts from acute injuries seem to facilitate fibroblast clearing and ECM degradation^141^.

Evidently, the intercommunication between fibroblasts and epithelial cells of the kidney is crucial for tubule regeneration after acute injury. A control of early fibroblast proliferation and clearance is also necessary, alike what happens in the lung. In opposition to other organs, the ECM in the kidney has not been studied in the context of regeneration.

### Heart

The heart does not regenerate after injury. Instead, lost cardiomyocytes are replaced by a fibrotic scar synthesized by fibroblasts that prevents ventricular wall rupture but contributes to functional decline^142,143^. Cardiac fibroblasts are uniformly dispersed throughout the interstitial space of the myocardium^142,144^ (Fig. 1f) and show a common embryonic lineage ancestry that can be traced to the endocardium and the proepicardial organ^145,146^. Multiple markers for cardiac fibroblasts have been reported (reviewed in refs. ^147,148^), but these are also found on perivascular cells and therefore two or more markers are normally combined to discriminate fibroblasts in the heart^149,150^.

In contrast to the adult mammalian heart, fetal and neonatal hearts regenerate following cardiac injury with minimal scarring^151^. In 2011, it was revealed for the first time that the neonatal heart of 1-day-old mice could regenerate after partial resection of the apex contrarily to as 7-day-old mice, which fail to regenerate their myocardium and develop significant fibrosis^152^. This regenerative response is therefore transient and relies on pre-existing cardiomyocyte proliferation^153^. Quaife-Ryan et al. compared all main cardiac populations (including CD90^+^ fibroblasts) in neonatal and adult hearts after myocardial infarction by RNA sequencing^154^, and found that adult fibroblasts are more responsive to injury than neonatal fibroblasts. Others show that neonatal fibroblasts display an intermediate phenotype between fetal and adult fibroblasts and contribute to the heart response to injury. Specifically, after neonatal cardiac injury, adult-like fibroblasts (PDGFRα^+^CD90^+^Sca-1^+^) exhibited increased expression of fibrotic-associated genes (*Col1a1*, *Col3a1*, *Tgfb1*, and *Tgfb3*), whereas fetal-like fibroblasts (PDGFRα ^+^CD90^+^Sca-1^−^) had also increased expression of genes associated with improved cardiomyocyte proliferation (*Fn1*, *Tbx20*, *Igf1*, *Igf2*, *Fstl1)* and neovascularization (*Vegfa)*^155^. A recent single-cell RNA-sequencing approach characterizing non-myocyte populations in regenerative and reparative neonatal mice hearts has unveiled subsets of fibroblasts, which respond differently to injury. Contrarily to regenerative hearts, in which the subsets remain fairly constant after injury, in non-regenerative hearts, proliferating and activated fibroblasts become more prevalent^156^. These findings support that cardiac fibroblasts diversity may comprise cell subsets with pro-regenerative or pro-reparative phenotypes.

Cardiac fibroblasts impart on the response to injury through the production of ECM. In fact, ECM from regenerative hearts displays different components when compared those of reparative hearts^157,158^. Fibronectin, periostin, and agrin have all been correlated to a pro-regenerative setting^16^. In zebrafish, fibronectin is required for regeneration by promoting fibroblast migration and cardiomyocyte proliferation^159,160^. Embryonic mouse fibroblasts also express high levels of fibronectin, collagen, and heparin-binding EGF-like growth factor, which promote mitotic activation in cardiomyocytes during late development^161^. Increased deposition of agrin and periostin upon injury in P1 neonatal mice has been related to cardiac regeneration by activating the phosphoinositide 3-kinase and Yes-associated protein signalling pathway, respectively, and subsequent induction of cardiomyocyte re-entry in the cell cycle^162–164^. Even though tenascin-C has been reported as a pro-regenerative component in lower vertebrates^165^, in mice there are contradictory evidences regarding its role in the heart response to injury. Tenascin-C is highly expressed in the infarct zone, and different studies indicate that promotes neonatal regeneration by attenuating inflammation and promoting cardiomyocyte proliferation^159,166^. On the other hand, tenascin-C can be a predictor of fibrosis since high expression of tenascin-C in a fibrosis model is correlated to collagen deposition in later stages^167^. Although no evidence has been shown in mammals, hyaluronic acid is another component of the ECM that can promote heart regeneration in newts and zebrafish by promoting cell migration^165^.

Apart from ECM modulation, cardiac fibroblasts can signal for regeneration through the secretion of growth factors and cytokines, both in the adult^168^ and the neonatal^156^. Although the precise involvement of fibroblasts in neonatal regeneration is undefined, different studies have accessed the role of fibroblasts and fibroblasts-produced molecules in the adult response to injury. Furtado et al. have suggested that fibroblast expressing cardiogenic markers are associated to a regenerative response after injury, as the loss of *Tbx20* expression hampered cardiac repair^169^. Fibroblasts express fibroblast growth factor 1 (FGF-1), which induces cardiomyocyte cell-cycle re-entry^170,171^. After myocardial infarction, an injection of FGF-1 in combination with p38 MAP kinase inhibition enhances the proliferation of both cardiomyocytes and endothelial cells, resulting in reduced scar and improved heart function^170,172^.

The discovery of the regenerative capacity of neonatal hearts has highlighted the importance of age in the response to injury. Since then, various studies have linked particular ECM components expressed in neonatal animals to regeneration, but the specific role of cardiac fibroblast in the cardiac regenerative response is poorly defined.

## Fibrosis vs regeneration: the impact of aging

Most tissues exhibit a progressive decay in their regenerative capacity from the onset of development to the end of their lifespan. In fact, aging leads to a decline in tissue function, reducing the ability of tissues to repair after damage and maintain homeostasis. The recently reported Tabula Muris Senis study has provided insight on cell dynamics and profiles in aging mice across all organs^173^. In tissues like the intestine, aging is associated to a decay of adult stem cell regenerative potential. However, the aging phenotype of stem cells can be rescued when exposed to a “young” environment. For example, the aged phenotype of muscle stem, adipose mesenchymal stem, and hematopoietic stem cells have been rescued after exposure to the blood of a younger animal using a parabiosis model^174^. As noted before, the reactivation of Wnt in the intestine is capable of rejuvenating aged ISC^32^. In other organs, it is known that aging promotes the development of progressive fibrosis and ECM deposition linked to a low-grade inflammation (inflammaging), leading to a hindered regenerative capacity in the kidney^175–177^, the lung^178,179^, the liver^180,181^, and the heart^182^. Indeed, the ECM of the majority of the organs shows age-dependent biochemical and mechanical modifications^104,182–185^, which suggests a great influence of fibroblast activity and dynamics on the behavior of neighboring cells. Alongside, the secretome of fibroblasts is gradually modified from development to aging, imparting differently on organ function and response to injury. In different organs, fetal and adult fibroblasts have been associated with pro-regenerative and pro-fibrotic phenotypes, respectively. In the skin, fetal dermal fibroblasts favor scarless wound healing by expressing high levels of FGFs and TGF-β1, compared to adult fibroblasts^186^. In the heart, the transcriptome and epigenome of fetal and adult cardiac fibroblasts have been compared showing that besides being smaller and proliferating faster, fetal fibroblasts express high levels of IL-8 signaling, ephrin receptor signaling, and Notch signalling pathways, compared to adults^187^. Recently, single-cell RNA-sequencing analysis of mouse hearts at different ages has unveiled that the transition from neonatal to adult fibroblast state directly supports cardiomyocyte maturation^188^. The same report showed that, in in vitro co-culture studies, adult fibroblasts reduce immature cardiomyocyte proliferation and promote their electrophysiological maturation. The composition of fetal/neonatal and adult fibroblast-derived ECM is also different and young ECM has shown to be a preferable environment to maintain cardiac cells, compared to the adult counterpart. In addition, the composition and stiffness of the ECM has been show to directly influence the regenerative capacity of the neonatal heart^189^. Collectively, this evidence supports that, in two organs with such different regeneration potentials, younger fibroblasts provide pro-regenerative cues when compared to adult fibroblasts.

Aging is often related to cellular senescence^190^. Several reports on fibroblasts and aging indicate that fibroblast senescence is one of the underlying causes of poor regenerative capacity and of the formation of extensive fibrosis. Compared to younger counterparts, aged dermal fibroblasts show features of cell senescence, such as senescence-associated β-galactosidase, are less responsive to TGF-β activation and migrate less, ultimately leading to slower wound healing^191^. In idiopathic pulmonary fibrosis, which is more common in the elderly, lung fibroblasts express more α-SMA, collagen I, and secrete high levels of inflammatory cytokines^192^. A report of induced senescence in the lung shows that lung fibroblasts have decreased expression of collagen I, elastin, and fibronectin, which are important for alveologenesis^193^. In the heart, aging fibroblasts are pro-inflammatory and express *Serpine1* and *Serpine2*, promoting age-associated endothelial dysfunction^194^. In the intestine, senescent fibroblasts secrete growth differentiation factor 15 (GDF15), a senescence-associated factor, which stimulates dysregulated epithelial cell proliferation, migration, and, ultimately, tumor formation^195^. On the other hand, other reports propose that cell senescence, occurring at least transiently, is key to limit fibrosis^196–198^. In the heart, senescent fibroblasts accumulate in the injury area after myocardial infarction and limit collagen production. Knocking down the senescence marker p53 resulted in reduced inflammation, which leads to the conclusion that cardiac fibroblast senescence is beneficial if limited in time^199^. In the liver, HS cell senescence and further clearing by immune cells is reported to limit fibrosis, with reduced secretion of ECM and increased ECM degradation^94^. Transient senescence after injury is beneficial by limiting fibrosis likely because it targets mostly myofibroblasts. This scenario contrasts with age-associated chronic senescence, in which homeostatic fibroblasts are the primary targets, and that ultimately leads to the loss of regenerative capacity, aberrant ECM deposition and organ fibrosis^200^.

**Fig. 2:**
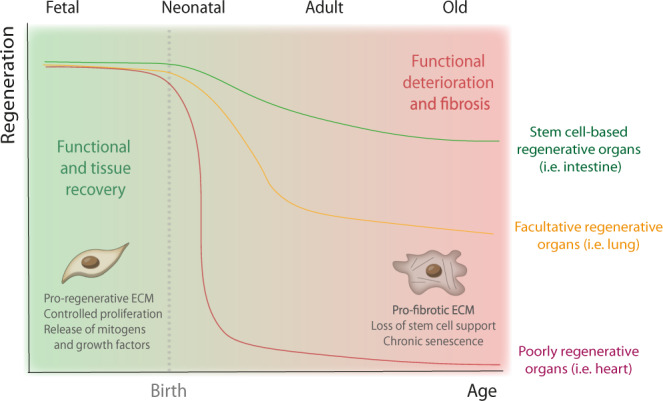
Correlation of the regenerative potential of organs with aging. Whilst less regenerative organs, as the heart, decrease their regenerative capacity abruptly after birth, regenerative organs, such as the skin and intestine, experience a progressive decline in their renewal capacity with increasing age. Organs like the lung depend on specific signals to mount a regenerative response—facultative regeneration—however, this ability is also impaired in the elderly. The loss of regenerative capacity in most organs seem to associate with the transition from a “young” to an “old” fibroblast phenotype. Whilst young fibroblasts are pro-regenerative and promote healthy tissue recovery, aged fibroblasts fail to support the regenerative niche, ultimately contributing to fibrosis and loss of organ function.

In sum, the axis of repair-regeneration mediated by fibroblast activity in different organs seems to be profoundly affected by age (Fig. 2). The younger the organism, the better fibroblasts maintain homeostasis and respond to injury in a pro-regenerative fashion. Older fibroblasts appear to be heterogeneous and respond with varying healing rates to reprogramming and wounding^201^. Senescence, typically associated to aging, may be beneficial to regeneration, but only if limited in time. Furthermore, evidence that most regenerative ECM components are typically expressed by fetal/neonatal fibroblasts supports the view that young ECM is a pro-regenerative environment whereas the adult/aged ECM supports pro-reparative/fibrotic responses. In fact, most pro-regenerative ECM-related molecules mentioned above decrease their expression with aging (Table 1). Overall, whilst the beneficial effect of fibroblasts in regeneration is emerging in different organs, most studies fail to demonstrate causality. In this context, future studies addressing the impact of fibroblast abrogation (e.g., through genetic models, as performed in the heart^202^) on the regenerative response will be important to provide comprehensive understanding on the role of these cells in organ renewal.

**Table 1:** ECM-associated molecules^207^ involved in the regeneration in in vivo studies of different organs and their transcriptional regulation with age.

	Organ	Function	Expression in aging	Model	Refs.
Core matrisome					
Agrin	Heart	Promotes CM proliferation in regenerative hearts	↓	Mouse	^163^
Chondroitin sulfate/aggrecan	Skin	Enhances wound closure in oral mucosa and in *Acomys*	↓	*Acomys*, Human	^63,208^
Collagens	Liver	Collagen I: may induce progenitor cell differentiation	N/A	Mouse	^101^
	Skin	Collagen III: favors cell migration in fetal wound healing	↓	Mouse, Rat	^209,210^
CTHRC-1	Skin	Promotes recruitment of M2 macrophages	N/A	Mouse	^64^
Elastin	Skin	Downregulated in oral mucosa	↑	Human	^208^
	Lung	Present in alveolar septation	↑	Mouse	^211^
Fibronectin	Skin	ED-A form: likely influences cell migration in oral mucosa	↓	Human, Rat	^208,212^
	Liver	Induces cell proliferation and progenitor differentiation	↓	Mouse	^101,213^
	Lung	Promotes endothelial cell adhesion	↓	Rabbit	^111,214^
	Heart	Favors fibroblast migration and CM proliferation in zebrafish	↓	Rat, Zebrafish	^160,215^
Hyaluronic acid	Skin	Promotes cell migration in scarless wound healing	↓	Human, Rabbit	^216,217^
	Heart	Supports cell migration	N/A	Newt	^165^
Laminins	Skin	Laminin α1: present in regenerative *Acomys* skin	N/A	*Acomys*	^63^
Olfactomedin	Liver	Promotes cell migration and adhesion	N/A	Mouse	^101^
Periostin	Intestine	Regulates epithelium proliferation	N/A	Human, Mouse	^34^
	Heart	Promotes fibroblast migration in P1 regenerative hearts	↓	Mouse	^218^
Tenascin-C	Skin	May help promote fibroblast and epithelial cell migration	↓	Human	^73,219^
	Heart	Influences newt CM proliferation in vitro	↓	Mouse, Newt	^165,220^
Vitronectin	Liver	Signals to increase in cell migration and proliferation	N/A	Rat	^101,221^
ECM affiliated proteins					
Semaphorin 3	Intestine	Supports epithelial growth	↓	Mouse, Rat	^21,222^
ECM regulators					
MMPs	Skin	Increased MMP/TIMP ratio in fetal wound healing supports matrix degradation	↓	Rat	^223^
	Liver	MMP-1, 2: promote ECM remodeling	=	N/A	^224^
Secreted factors					
Angiopoietin	Liver	Promotes angiogenesis	N/A	Rat	^225^
Epimorphin	Liver	Stimulates hepatocyte proliferation	N/A	Mouse	^226^
Erythropoietin	Liver	Promotes angiogenesis	↓	Human	^227^
FGF-1	Heart	Induces CM cell-cycle re-entry	N/A	Mouse	^171^
HGF	Intestine	Promotes cell proliferation	N/A	Mouse	^21^
	Liver	Promotes cell proliferation	N/A	Mouse	^228^
	Kidney	Promotes cell proliferation and TGF-β antagonization	N/A	Mouse	^137^
Pleiotrophin	Liver	Stimulates hepatocyte proliferation	N/A	Rat	^229^
Shh	Skin	Promotes hair follicle maintenance	↓	Mouse	^72,230^
VEGF	Liver	Promotes angiogenesis	↓	In vitro rat HS	^231,232^
Wnt	Intestine	Promotes stem cell niche maintenance	↓	Mouse	^26,29,32^
	Skin	Promotes stem cell niche maintenance	↓	Mouse	^71,233^

*N/A* information not available.

## Concluding remarks

Fibroblasts remain the main culprits of fibrotic diseases in most organs. The phenotypic heterogeneity of these cells is stressed by the observation that fibroblast markers in different organs mostly do not overlap. Yet, PDGFRα is present in resident fibroblasts from most organs. PDGFRα^+^ fibroblasts can differentiate into adipocytes and myofibroblasts in various organs, such as the lung and the heart^113,203^. All reported fibroblast markers also do not distinguish pro-fibrotic from pro-regenerative populations across organs and within the organ itself, although recent reports on synovial fibroblasts suggest the existence of discrete subpopulations of fibroblasts with opposite functions based on the expression of a single marker^204,205^. In the future, data collected from single-cell RNA-sequencing studies across organs will be of utmost importance to define the signature of homeostatic, pro-fibrotic, and pro-regenerative fibroblasts.

Apart from the well-known role of fibroblasts in tissue ECM maintenance in homeostasis and remodeling after injury, a new perspective is emerging on the pro-regenerative role of resident fibroblasts. In this context, they may act in a cell-intrinsic manner or by modulating the microenvironment of the stem cell niche, when existing, by: (i) producing matricellular components, namely growth factors or mitogens, (ii) activating signaling pathways (e.g., Wnt, Hedgehog, retinoic acid), (iii) proliferating in early and restricted time-period of injury, or (v) creating the scaffold that guides regeneration and immunomodulation.

Importantly, fibroblast-mediated regeneration seems to rely on the activation of embryonic/neonatal gene expression programs across different organs. Based on this evidence one can hypothesize that reverting the adult fibroblast/myofibroblast phenotype to a fetal or neonatal stage could be a promising therapeutic avenue that can be applied to different organs. Recently, a proof-of-principle study showed that in vitro rejuvenation of fibroblasts is achievable and reverted the fibroblast aging phenotype, which can be useful for regenerative medicine purposes^206^.
