# Author Correction: The bright side of fibroblasts: molecular signature and regenerative cues in major organs

**DOI:** 10.1038/s41536-023-00319-x

**Published:** 2023-08-07

**Authors:** Rita N. Gomes, Filipa Manuel, Diana S. Nascimento

**Affiliations:** 1 Instituto de Investigação e Inovação em Saúde (i3S), Universidade do Porto, Porto, Portugal; 2 Instituto de Ciências Biomédicas Abel Salazar (ICBAS), Universidade do Porto, Porto, Portugal; 3 Instituto Nacional de Engenharia Biomédica (INEB), Universidade do Porto, Porto, Portugal; 4 Faculdade de Ciências, University of Lisbon, Lisbon, Portugal

**Keywords:** Mechanisms of disease, Ageing, Diseases

Correction to: *npj Regenerative Medicine* 10.1038/s41536-021-00153-z, published online 10 August 2021

In the Acknowledgements section, the funding source ‘This work was funded by European Structural and Investment Funds (ESIF), under Lisbon Portugal Regional Operational Programme and National Funds through Fundação para a Ciência e Tecnologia (FCT) ([POCI-01-0145-FEDER-030985 and POCI-01-0145-FEDER-031120] and);’ should have read ‘This work was funded by the European Regional Development Fund (ERDF) through COMPETE 2020, Portugal 2020 and by FCT (Fundação para a Ciência e Tecnologia, ([POCI-01-0145-FEDER-030985 and POCI-01-0145-FEDER-031120] and);’. The original article has been corrected.

